# Accelerating biosimilar market access: the case for allowing earlier standing

**DOI:** 10.1093/jlb/lsae030

**Published:** 2025-01-03

**Authors:** S Sean Tu, Rachel Goode, Matthew Turner, Victor Van de Wiele

**Affiliations:** West Virginia University College of Law, 101 Law School Dr, Morgantown, WV 26506, USA; Fresenius Kabi Biopharma, Terre Bone Business Park, Route de Crassier 23, Eysins, 1262 Vaud, Switzerland; Fresenius Kabi Biopharma, Terre Bone Business Park, Route de Crassier 23, Eysins, 1262 Vaud, Switzerland; Division of Pharmacoepidemiology and Pharmacoeconomics, Brigham and Women’s Hospital, 1620 Tremont St. Suite 3030, Boston, MA 02120, USA

**Keywords:** patents, medicare, biologic, biosimilar, drug spending

## Abstract

Biosimilars, which are affordable alternatives to biologic medicines, face delays in market entry due to the current patent litigation framework under the Biologic Price Competition and Innovation Act. Currently, biosimilar manufacturers can only initiate patent litigation to attempt to clear weak and invalid patents after submitting their Biologic License Application to the Food and Drug Administration (FDA), which happens after completing extensive, and costly clinical trials. By contrast, generic drug manufacturers can start litigation earlier due to shorter development times and less stringent clinical requirements, allowing them to launch immediately after the primary patent expires. We propose allowing biosimilars to begin patent litigation at the start of phase 3 clinical trials, the final stage of biosimilar development, where the product and manufacturing process and product profile are largely finalized. This change would enable biosimilar firms to resolve patent issues well before the brand biologic’s primary patent expiration date, potentially reducing market entry delays by about 1.8 years. This article examines the issues surrounding initiation of biosimilar litigation and suggests litigation reforms to expedite biosimilar market availability.

## I. INTRODUCTION

From 2015 to 2021 there were only 11 biosimilars marketed in the US. In contrast, from 2008 to 2021, there were 55 biosimilars marketed in in the European Union.^1^ This disappointing US output occurred despite the $261 billion in revenues that biologics generated in 2021, which comprised 46 per cent of all US drug spending.^2^ Although biosimilar approvals have recently increased in the US, delayed entry persists. Biosimilars have the potential to drive these costs down with projected savings created by biosimilars to exceed $180 billion over the next five years.^3^ Accordingly, Congress should promote strategies to expedite biosimilar market entry, while balancing the brand biologic’s need to recuperate costs and make enough profits to incentivize new drug innovation.

Biosimilar firms (hereinafter ‘biosimilars’) may not enter the market when there are relevant, valid patents covering the drug, regardless of the quality of those patents. Patents can cover the active ingredient (primary patents) or follow-on innovation (secondary patents), such as methods of use, methods of manufacture, formulation, route of administration, and sometimes even the devices used to administer the drug.

Primary patents typically are the strongest patents and are the most difficult to invalidate or design-around.^4^ Typically, generic drugs and biosimilars must wait until the primary patents expire before market entry. By contrast, secondary patents can extend monopoly rights long after the primary patents expire, but still must be challenged and revoked before biosimilars can enter the market free from risk. Secondary patents are invalidated at a higher rate and are generally considered weaker than primary patents.^5^ For example, biosimilar firms that challenge the validity of biologic patents were successful in invalidating at least one claim in 70 per cent of all instituted America Invents Act-challenged patents, and most of these patents were secondary patents.^6^ Without this type of ‘patent clearance’ the biosimilar risks an injunction that would prevent market entry or monetary damages owed to the patent owner.^7^

Eliminating invalid patents is important because all relevant patents must be addressed before a biosimilar can enter the market without risk. Without patent pre-clearance, lower cost biosimilars may not enter the market in a timely fashion. One way to evaluate efficient biosimilar market entry is by examining the legal framework for challenging biologic patents, specifically litigation initiation under Biologic Price Competition and Innovation Act (BPCIA).

### I.A. Differences between Biosimilar and Small-Molecule Market Entry Requirements

There are two broad categories of new drugs: small-molecule drugs and biologic drugs. Generic drugs are copies of branded small-molecule drugs while biosimilars are copies of branded biologic drugs. Small-molecule drugs are low-molecular weight products derived from chemical compounds, while biologics are high-molecular weight products produced by living cells in a bioreactor or fermenter.^8^ Small-molecule drugs are chemically synthesized and are relatively easy and inexpensive to produce. By contrast, biologics are more complex in nature, and the methods of manufacture need to be tightly controlled to ensure consistent purity, potency and safety.

The cost of biosimilar development is much greater than generic drug development. Generic drug development typically costs $3 million and takes ⁓3 years. By contrast, the development of a biosimilar typically costs over $150 million and takes over 8 years.^9^^,^^10^ Biosimilar development costs are greater in part because of the high price of the reference product (needed to be purchased for comparison studies at list price), the complex nature of manufacturing the biosimilar product, and conducting expensive clinical trials.

Generic manufacturers do not need to conduct lengthy phase 3 clinical trials to obtain FDA approval for marketing because the small-molecule active ingredient is identical to the brand drug. Under the Hatch-Waxman Act, the patent litigation process starts when a generic firm files its Abbreviated New Drug Application (ANDA) with the FDA.^11^ Because generic manufacturers do not have to engage in lengthy phase 3 trials, they often submit their ANDAs long before primary patent expires.^12^ This allows the generic manufacturer to engage in patent litigation to clear secondary patents and complete the litigation process before the primary patent expires. By clearing a path through these secondary patents, generic manufacturers can launch their products close to the primary patent’s expiration date.^13^

Biosimilar development cannot begin until after FDA approval of the brand biologic’s product, which the biosimilar firm must use as a reference product. This delay in development is created, in part, because the biosimilar firm requires samples of the reference product for analytical testing to determine the precise profile (for example, glycosylation profile, oxidation profile, etc.) of the drug to be copied.^14^ These samples are not available until the brand biologic launches their product and makes it available for commercial purchase.

Additionally, biosimilar firms must conduct costly and lengthy phase 3 trials to show that their products are similar in safety, purity and potency. The typical biosimilar phase 3 trial takes ⁓4.6 years to complete, and biosimilar firms cannot initiate litigation under the BPCIA until after phase 3 trials are completed^15^. The lengthy development programs of biosimilars leads to the litigation of secondary patents *after* expiration of the primary patent. This stands in contrast to generic drug patent clearance timelines, where secondary patent litigation occurs before the primary patent’s expiration date to allow for market entry quickly after the primary patent’s expiration date.

### I.B. The Current Biosimilar Litigation Framework

A biosimilar firm has three options for entering the market. First, they could pre-clear the patents before entering the market by engaging in the ‘patent dance’ and associated patent litigations. A second option is to launch ‘at risk.’ Finally, biosimilar firms could settle with the brand biologic to avoid any active patents.

#### I.B.1. The Patent Dance and Biosimilar Patent Litigation

Biosimilar patent litigation is governed by the BPCIA which includes a pre-litigation process known the ‘patent dance’.^16^ The patent dance is triggered when the biosimilar submits its FDA approval application and includes an exchange of information with the goal to provide clarity as to which patents may be relevant for litigation.^17^

Following the patent dance, the brand biologic then files a patent infringement complaint, asserting the patents listed by both parties in the patent dance.^18^ The complaint initiates a traditional patent litigation procedure called ‘the first wave’ of litigation. The ‘second wave’ of litigation is triggered when the biosimilar firm gives 180 days’ notice to the brand biologic of its intent to begin commercial marketing.^19^ Similar to the first wave of litigation, the second wave litigation cannot begin earlier than submission of the biosimilar application to the FDA. During the second wave litigation, the brand drug company is free to assert patents that the biosimilar firm did not agree to include during the patent dance. At this time, the branded drug company can also request a preliminary injunction to stop the biosimilar from manufacturing or selling its product. The purpose of requesting a preliminary injunction is to try to maintain the brand biologic’s market exclusivity until the completion of patent litigation.

Only after both waves of litigation are resolved, will the biosimilar firm know if it can launch its product free from patent risk. If litigated to final conclusion, firms have to engage in the first and second waves of litigation and the inevitable appeals associated with these litigations. Litigation can last for several years following FDA approval of the biosimilar, leading to delayed launch of the biosimilar long after the expiry of the primary patent.

#### I.B.2. Launching At Risk/Settlement

A biosimilar firm can launch ‘at risk’ which means it launches the biosimilar product while patent litigation is still ongoing. Launching at risk poses a significant threat to biosimilars because infringement findings could lead to ‘lost profit’ damages totaling hundreds of millions, well surpassing the anticipated earnings from the biosimilar product. This intimidating remedy imposes a difficult decision on biosimilar firms whose patent litigation is pending at the time they receive FDA approval for their product.

Alternatively, the biosimilar may settle the patent litigation with the brand drug company and agree to a market entry date. We find that most biosimilar firms enter into settlements with the brand biologic to resolve the risk associated with potential patent infringement and oftentimes to reduce the delay associated with awaiting a final decision from the court on the litigation. Nevertheless, patent settlements usually involve launching with some delay after both FDA approval of the biosimilar and following expiry of the primary patent.

## II. SHIFTING LITIGATION FOR EXPEDITED BIOSIMILAR MARKET ENTRY

One way to help biosimilars enter the market in a timely fashion, similar to generic drugs, is to allow patent litigation to start earlier. Specifically, biosimilars could be allowed to challenge patents at the beginning of the phase 3 clinical trials. Allowing biosimilars to bring suit earlier would help them enter the market when the primary patent expires, which would parallel the generic market entry process.

There are several hurdles biosimilars may encounter if allowed to bring a lawsuit earlier in the biosimilar development process. First, the judicial ‘ripeness’ doctrine, may apply to prevent earlier initiation of the litigation process. Second, branded drug companies may consider that earlier litigation may be premature if the biosimilar product or manufacturing process is changed during or after phase 3 trials. Third, if the biosimilar does not succeed in completing phase 3 trials, then initiating the litigation process at an earlier point in time would have brought the brand firm into unnecessary, expensive litigation and waste court resources.

### I‌I.A. Judicial Ripeness

Before a court can take a case, the issues must be ‘ripe.’ A case is not ripe for adjudication if it rests upon contingent future events that may or may not occur.^20^ In assessing ripeness, courts will consider ‘how far in the future the potential infringement is, whether the passage of time might eliminate or change any dispute and how much if any harm the potential infringer is experiencing, at the time of the lawsuit, that a litigation might redress.’^21^

In Sandoz v. Amgen, the court was asked to assess the ripeness doctrine in a biosimilar patent litigation. Sandoz, the biosimilar firm, sought to challenge the validity of the patents with respect to their etanercept biosimilar. Because the BPCIA did not include a pathway for Sandoz to initiate a litigation prior to submitting their FDA approval application, Sandoz filed a declaratory judgment action three years prior to obtaining FDA approval. The district court concluded that the case was not ripe and no Article III controversy existed between the parties. The court reasoned that the BPCIA established the procedures for narrowing and resolving patent disputes between biosimilar and brand biologic firms and that Sandoz could not obtain a court judgment before filing an FDA application.^22^ The Court of Appeals of the Federal Circuit (CAFC), the sole appellate court for patents, affirmed this decision. The CAFC concluded that a biosimilar firm ‘cannot engage in…liability-exposing conduct…without FDA approval of an application precisely defining the products it may market.’^23^

The court expressed concern that the phase 3 clinical studies might fail, potentially resulting in Sandoz not filing for FDA approval, thereby eliminating the patent dispute and wasting court resources. The court was also concerned that the biosimilar firm might have to modify its proposed product prior to submitting its FDA application, which might alter the list of patents in dispute. The court concluded that anything earlier than submission of the FDA application would be too premature to litigate because the case would not be ripe.

This case was decided in 2014, and at the time, no biosimilar had completed the FDA approval process. The CAFC admitted that ‘[t]he biosimilarity approval standard is new; indeed, the FDA has not yet applied the new standard to complete its review of and approve any product under the BPCIA…[and] [*p]erhaps the FDA is exercising a caution that will prove excessive over time. But we have no basis for saying so*.’^24^

In light of a decade of additional biosimilar approvals, we find that the court’s concerns were unfounded. We find that pivotal phase 3 clinical studies require that the manufacturing process to be sufficiently fixed, preventing biosimilar firms from making significant changes to their product or manufacturing methods afterwards. Therefore, initiating litigation at the start of phase 3 trials would not be premature. Additionally, we find that most biosimilars that enter into phase 3 studies result in FDA-approved biosimilars.^25^ Therefore, litigation at this stage will neither result in unnecessary litigation nor waste court resources.

### I‌I.B. Litigation Would Not be Premature if Initiated at the Start of Phase 3 Clinical Studies

The process of manufacturing a biosimilar is crucial since the method of manufacture contributes the final biosimilar product profile. For example, the primary amino acid sequence, cell culture media and bioreactor conditions determine the ultimate physiochemical properties of a biosimilar product, including its glycosylation profile. Equally, steps used in the purification process of the biosimilar product influence its charge profile and purity profile.

Extensive process development and product characterization are required *before* entering phase 3 trials and are expected to be ‘locked’ before the drug is administered in human trials. This is because the FDA requires that the proposed biosimilar used in the phase 3 study be representative of the to-be-marketed biosimilar.^26^ Changing the product or process after beginning the phase 3 clinical study is undesirable because biosimilar firms would likely need to generate extensive additional comparability data and/or additional clinical trials, adding significant delays and costs on their development program.^27^ Therefore, biosimilar firms are already incentivized to avoid changing their product or process in a manner that could impact the patent dispute.

### I‌I.C. Biosimilar Phase 3 Trials Are Unlikely to Fail so Litigation Should Not be a Waste of Court Resources

Brand drug companies may be concerned that, should the biosimilar not succeed in phase 3 trials, then initiating the litigation process at this time point would have brought the brand firm into unnecessary and expensive litigation. However, we find that a low percentage of biosimilars fail to obtain FDA approval following initiation of their phase 3 clinical study. This is largely because biosimilar phase 3 clinical trials are almost always confirmatory, meaning that the clinical study is unlikely to fail.

It is noteworthy that biosimilar phase 3 studies are far more likely to succeed as compared to branded biologic phase 3 studies. This is because brand biologics use phase 3 studies to demonstrate the risk and benefit of an *untested* drug candidate and large phase 3 trials that are required to show efficacy and safety. By contrast, the purpose of a biosimilar phase 3 trial is rather to *compare* the biosimilar to the brand biologic. By the time the biosimilar reaches the phase 3 trial stage, it has already undergone extensive evaluation and is advancing steadily towards obtaining FDA approval, hence the phase 3 study is merely confirmatory.

Prior to the phase 3 study, the biosimilar firm is required to build a robust package of data including considerable, highly sensitive, analytical testing that compares the product profile of the biosimilar to the branded drug. All this information could be used by brand biologics to determine which product and process patents might be infringed.

Further evidence that biosimilar phase 3 studies are primarily confirmatory in nature comes from the regulatory field, where considerable debate exists over whether biosimilar clinical trials are even needed at all.^28^ The Biosimilar User Fee Act III states that the FDA may waive and omit phase 3 studies for biosimilars. Similarly, the European Medicines Agency (EMA) is considering phase 3 study waivers. EMA-associated scientists reviewed all approved biosimilars and concluded that patient-trials did not play a decisive role in decision making.^29^ Others argue that there is enough evidence from analytical, functional and pharmacokinetic studies to show biosimilarity.^30^ The UK regulatory agency, already provides an option for biosimilars to waive phase 3 studies. These regulatory changes all suggest that biosimilar phase 3 studies are confirmatory in nature, meaning that biosimilar development can be considered more or less finalized once it reaches the stage of phase 3 clinical studies.

To investigate the obstacles associated with earlier litigation, we examined the failure rate of biosimilar approval beginning at the phase 3 trial stage. Additionally, we assessed the current timing of litigation to estimate potential time savings if litigation was initiated at an earlier stage.

## III. METHODS

### I‌II.A. Patent Litigation Data Sources

We used the Lex Machina litigation database to identify all patents involved in biosimilar litigation from January 1, 2010, the year that the biosimilars pathway was enacted, to December 31, 2022. The corresponding complaint date, district court termination date and CAFC termination date was identified for each biosimilar.^31^ Lex Machina also classifies each litigation as: likely settled, open case, claimant win, or claim defendant win. For those cases that were litigated to final judgment, we reviewed the opinion to determine if the brand biologic or biosimilar firm ‘won’ the case. For cases that settled we used the court case dismissal date as the settlement execution date. A win for the brand biologic typically resulted in an injunction from making or using the product and could include monetary damages for lost profits. A win for the biosimilar typically resulted in either invalidation of the patent or a non-infringement decision.

We assessed the timing of each patent litigation, when the litigation was initiated, the time taken to reach a first instance court decision, and a final decision on appeal. Appellate decisions from the CAFC were analyzed using Lex Machina. We used the median length of litigation as a unit for extrapolation (complaint to final judgment/dismissal). We also assessed the cases settled before a court decision was reached. We compared the timing of the patent litigation or settlement, as compared to when the biosimilar received FDA approval, the date of expiry of the primary patent, and the date of biosimilar launch. If the court prevented biosimilar entry until a specific injunction date, we used the injunction date as the predicted launch date.

For each litigated biosimilar we identified the: clinical trial ID, clinical trial start date, and clinical trial end date.^32^ The biosimilars FDA approval application and FDA approval date were identified.^33^^,^^34^

For biosimilars that litigated to final judgment that resulted in a successful challenge (n = 5) or failed challenge (n = 3), we determined the median length of litigation (complaint date to appellate court final decision). For biosimilars that settled litigation (n = 24), we determined the median length of litigation (complaint date to district court dismissal date).

For all litigated patents, we reviewed each patent^35^ and determined if the patent was a primary patent, directed to the biologic’s active ingredient, or a secondary patent, directed to follow-on inventions (such as new formulations, methods of treatment, devices, etc). We calculated each patent’s expiration date and compared it against the settlement date or final court decision.

### I‌II.B. Clinical Trial Data Sources

We used PharmaProjects^36^ to collect data on biosimilar drugs (clinical and marketed) with phase 3 clinical trial studies conducted or partially conducted within the United States that measure biosimilarity with a brand biologic reference product. We collected the following data points: biosimilar name, reference product, and manufacturer. Rationales for phase 3 trial discontinuation were recorded. For example, if press releases stated that the trial was discontinued for commercial reasons or due to biosimilar bankruptcy.

We subsequently used clinicaltrials.gov^37^ to attain more specific phase 3 trial information, such as trial primary objectives. We took the earliest phase 3 trial and extracted: start date, end date, clinical trial number. We reviewed each clinical trial to determine if it was the pivotal trial relied upon by the FDA to grant biosimilar approval.

The median time from phase 3 to FDA approval for all biosimilars that received FDA approval from January 1, 2010 to December 31, 2022 was 4.7 years (IQR 3.45–5.9). Accordingly, when assessing whether a biosimilar may have failed to obtain FDA approval, we excluded those biosimilars that were less than three years after initiation of phase 3 trials (phase 3 submitted after April 1, 2021) because those biosimilars were likely still undergoing development prior to FDA approval. Because the median time between the start of phase 3 to approval was 4.7 years, the median time from phase 3 to FDA approval is 4.7, to favor the counter-viewpoint, we classified as failures those phase 3 trials that were submitted before April 1, 2021 (more than 3 years) and lacked information on clinicaltrials.gov.

For those biosimilars that completed a phase 3 trial, we included studies where the: (i) biosimilar launched and (ii) biosimilar engaged in patent litigation. We excluded those biosimilars that did not enter the market due to: (i) publicly declared commercial motivations, or (ii) if the clinical trials were likely still ongoing (if the phase 3 trial start date was after April 1, 2021).

### I‌II.C. Biosimilar Approval and Launch Data Sources

We used Drugs@FDA to extract biosimilar approval dates, biosimilar press releases, investor documents and Goodwin Procter LLP’s Big Molecule Watch to extract commercial launch dates of biosimilars.^38^

## IV. RESULTS

### IV.A. Phase 3 Clinical Trial Results

**Figure 1:**
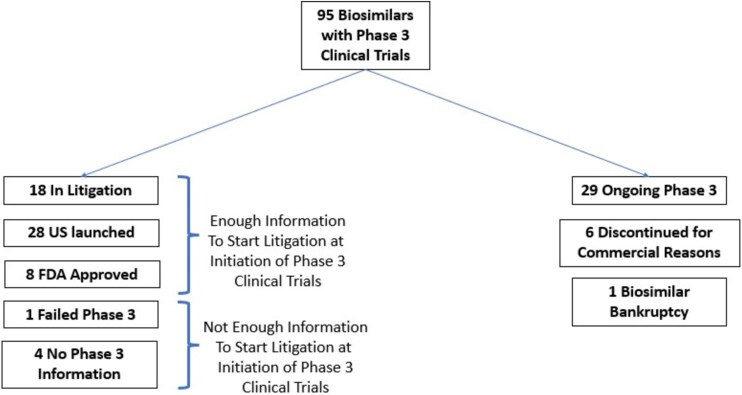
Determination of Study Cohort of Biosimilars with Phase 3 Clinical Trials from 2010 to 2022.

Among the 59 biosimilars that initiated phase 3 trials, 54 (92 per cent) resulted in enough information to begin litigation without wasting court resources. From 2010 to 2022, we identified 95 biosimilars associated with 25 brand biologics that reached the development stage of initiating phase 3 trials. When determining how many biosimilars may have failed to obtain FDA approval after initiating phase 3 studies, among the 95 biosimilars that started phase 3 trials, we included 59 and excluded 36. The reason we excluded 36 biosimilars: 29 (81 per cent) likely had ongoing clinical trials (filed less than 3 years from April 21, 2021), 6 (17 per cent) publicly declared as discontinued for commercial reasons, and 1 (3 per cent) biosimilar bankruptcy (Fig. 1).

 Among the remaining 59 biosimilars that initiated phase 3 trials, 36 (61 per cent) had launched in the US, 18 (31 per cent) were undergoing litigation, meaning they had submitted their aBLA application to the FDA, 1 (2 per cent) failed phase 3 trial, and 4 (7 per cent) provided no online information (filed before April 21, 2021).

We counted the four biosimilars for which there was no online information as failures, so as to bias the results towards the counter argument. In summary, among the 59 included biosimilars, 54 (92 per cent) went on to at least submit their dossier to the FDA, meaning court resources would not have been wasted if patent litigation had started at the initiation of phase 3 clinical trials. By contrast, only 1 (2 per cent) would have resulted in unnecessary litigation, with four additional cases (7 per cent) that *could potentially* result in unnecessary litigation costs and resources.

### IV.B. Litigation Results

From 2010 to 2022, 32 biosimilars entered the market with completed judicial proceedings. Five (16 per cent) of the 32 litigated biosimilars won litigations against the brand biologic, with 4 litigations appealed to the CAFC [3 affirmed; 1 procedurally dismissed]. Only 3/32 (9 per cent) of biosimilars lost litigation, with 2 being appealed to the CAFC [2 affirmed]. The remaining 24/32 (75 per cent) biosimilars settled and none appealed to the CAFC (all settlements occurred at the district court level).

The median duration for litigation (from the filing date of the complaint until a final court decision) is 2.9, 0.95 and 4.2 years, for biosimilar wins, settlements, and biosimilar losses, respectively (Table 1). Biosimilar launch is delayed substantially following a litigation loss because a permanent injunction is typically granted until primary patent expiry. Biosimilar market entry occurs a median of 2.3, 3.2, or 16.5 years after the primary patent expiry when biosimilars win, settle or lose litigation, respectively. Biosimilar market entry occurs a median of 1.3, 1.8, 10.0 years after initiation of litigation when biosimilars win, settle, or lose litigation, respectively.

**Table 1:** Median Duration of Key Litigation / FDA Events.

**Time Between**	**Biosimilar Win (years)** **n = 5**	**Settlement (years)** **n = 24**	**Biosimilar Loss (years)** **n = 3**
Phase 3 to Biosimilar Launch Date	3.6	5.7	15.9^*^
Phase 3 to FDA Approval	3.3	4.4	6.0
Phase 3 to Complaint	2.7	3.9	3.3
Phase 3 to Final Court Decision	5.9	4.6	7.6
Phase 3 to Primary Patent Expiration	2.8	3.0	−0.21
Complaint to Final Court Decision	2.9	0.95	4.2
Complaint to Biosimilar Launch Date	1.3	1.8	10.0^*^
Primary Patent Expiration and Biosimilar Launch Date	2.3	2.5	16.5^*^

^*^For biosimilar losses, estimated biosimilar launch dates were determined by the injunction date set by the court.

Most biosimilar patent cases are settled before a final judgment on the merits is reached. If a biosimilar settles the case, the litigation is resolved in 0.95 years after initiation of litigation. Following settlement, the biosimilar launch occurs a median of 1.8 years after initiation of the patent litigation. The median duration between the primary patent expiry and biosimilar launch is 2.3, 2.5, and 16.5 years when biosimilars win, settle or lose litigation. The median time between the start of phase 3 and primary patent expiration is 2.8, 3.0, −0.21 years when biosimilars win, settle, or lose. We note that when biosimilars lose litigation, they typically start their phase three trials after the primary patent expires. Therefore, we find that settlement allows biosimilars to enter the market earlier than if they had awaited the median duration of time for litigation to conclude (2.9 years or 4.2 years).

**Figure 2:**
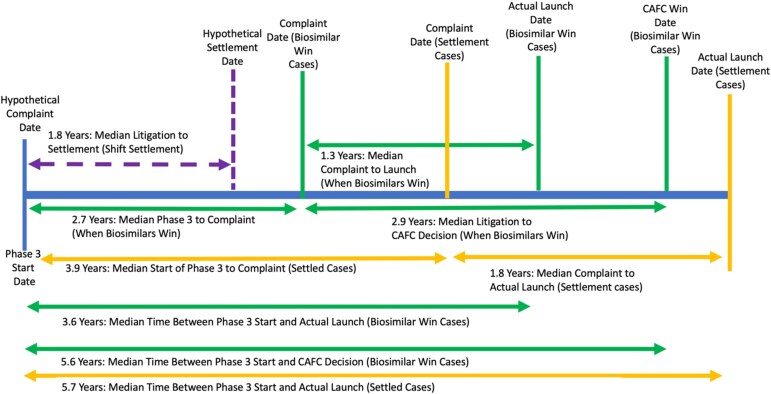
Timeline for Litigation (Medians).

**Figure 3:**
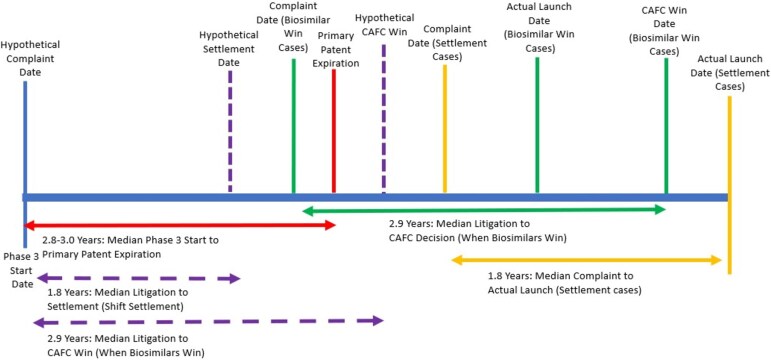
Timeline for Litigation (Medians) Compared to Primary Patent Expiration.

Allowing firms to initiate litigation at the start of phase 3 trials would allow firms to launch closer to the primary patent expiration date. For those firms with strong invalidity / non-infringement positions who litigate to final judgment, the appellate litigation would conclude about 1 month after the primary patent expires (Fig. 2). For those firms who decide to settle, shifting the litigation start time would allow biosimilars to enter close to the primary patent expiration date because litigation would terminate before the primary patent expires (Fig. 3).

## V. DISCUSSION

When biosimilars win patent litigation or settle, market entry occurs ⁓2.5 years after the primary patent expires. This is in stark contrast to generic drug entry where market entry occurs close to the expiration of the primary patent.^39^ By allowing biosimilar firms to start litigation at the start of phase 3 trials, they could litigate all relevant patents so that market entry occurs close to the expiration of the primary patent. Allowing biosimilar firms to litigate earlier will not be a waste of court resources because 91 per cent of all biosimilar firms who start phase 3 have sufficient information about their product and process to make the issue ripe for adjudication. Allowing biosimilar patent litigation to start upon initiation of phase 3 studies would provide more time to litigate the case prior to expiry of the primary patent, which would allow for earlier path clearing and may accelerate biosimilar entry.

Starting biosimilar litigation earlier may result in reducing the number of cases that settle. Currently, 75 per cent of biosimilars settle their cases with the brand drug company. From the biosimilar’s perspective, settlement removes the risk of being ordered to pay damages to the brand or being removed from the market with an injunction, eg, if they are eventually found to infringe secondary patents. From the brand’s perspective, if their primary patent has already expired, they may be concerned that a biosimilar would launch even while secondary patents are still in force. The uncertainty on both sides exists so long as the litigation is pending. However, earlier initiation of litigation may allow the patent landscape to be clarified in time for the biosimilar’s typical target launch date: expiry of the primary patent.

The median duration for litigation from the date the complaint was filed until a final court decision is 2.9 for biosimilar wins and 4.2 years for biosimilar losses. In comparison, the median time from phase 3 to FDA approval was 4.7 years (IQR 3.45–5.9). Therefore, we assume that almost all biosimilars would have sufficient time to complete their patent litigation before FDA approval if the litigation is initiated at the beginning of the phase 3 studies.

We find that 92 per cent of biosimilars progress to submitting an application to the FDA after initiation of phase 3 study while only 8 per cent fail. Therefore, allowing earlier litigation would not result in wasted court resources. This aligns with the fact that biosimilar phase 3 studies are merely confirmatory in nature and a robust comparability data package is already complete by the time the biosimilar reaches this last stage in development. Furthermore, allowing litigation to begin at this earlier time point is unlikely to be premature. When a biosimilar initiates phase 3 clinical trials, they are incentivized to ensure that their product profile does not change in a way that would alter any patent dispute. Changing the product or process after starting the phase 3 clinical study is undesirable to the biosimilar firm because it would typically be required to produce extensive additional comparability data and/or additional clinical trials, which would add delay and cost to the biosimilar development program.

Biosimilars must weigh three options: (i) the possibility of winning litigation resulting in launching 2.3 years after primary patent expiry; (ii) settling with the brand biologic resulting in an end to litigation and a launch delay of 2.5 years after primary patent expiry; or (iii) the possibility losing litigation resulting in a 16.5-year delay after primary patent expiry plus large damage awards. We conclude that biosimilars are likely making rational decisions when entering settlement agreements that allow them to launch earlier than awaiting the end of litigation and exposing them to large damage awards. We postulate that the settlement rate for biosimilars is higher compared to generics because biosimilar firms do not have timely access to a final court decision on the follow-on patents prior to expiry of the primary patent.

Several limitations of the present study require consideration. Because firms do not typically report failed clinical trials to clinicaltrials.gov, we may be overcounting failed trials because we include trials older than 3 years with no information as failed. We have also limited the phase 3 trials to those conducted and partially conducted in the United States. Although almost all of our data covers the key pivotal trials needed for biosimilar approval, there may be additional pivotal trials conducted in other countries. Finally, the BPCIA was enacted in 2010, and as a result, the quantity of biosimilars that have engaged in patent litigation and entered the market is limited.

### V.A. Possible Solution: Allow Biosimilars to Start BPCIA Litigation at the Start of Phase 3 Clinical Trials

We suggest that Congress create a two-track litigation system. Track One would be identical to the current BPCIA system that is already in place. This would allow biosimilar firms who are uncomfortable with the new system or who do not wish to invest in patent litigation until closer to the point of FDA approval to use a litigation procedure that occurs at the end of the drug’s development process. For example, this track may be selected by those few biosimilars who would anyway have launched at risk. Importantly, this proposal would not affect those firms who decide not to engage in the patent dance.

A second track, ‘Track Two,’ should be created to allow biosimilars to initiate patent litigation at the start of phase 3 trials. Track Two would shift the start of litigation from submission of the FDA approval application to the beginning of phase 3. Once the biosimilar enters this track, the biosimilar could bring a declaratory judgment action for invalidity and non-infringement of the branded patents and the patent owner could counter-sue. During the discovery part of litigation, the brand would have access to the biosimilars manufacturing process, product profile information and other confidential information, as per a traditional patent litigation. The brand would then include all potentially relevant patents into the lawsuit or be precluded from asserting them in future litigation. Similar to the 3A list of the BPCIA patent dance, following disclosure, no additional patents could be added to the litigation. Track Two would follow a traditional litigation process and would not include the patent dance, nor the first/second waves of litigation provided for under the BPCIA. The brand biologic, however, would be stopped from bringing later litigation based on patents not litigated unless the biosimilar manufacturing process or product profile changes to render additional patents relevant.

Like the current BPCIA framework, the biosimilar firm would have to provide the brand biologic with a copy of their FDA dossier as soon as it becomes available and provide a 180-day notice of commercial marketing, even if the Track Two litigation is already complete. This would allow the brand biologic a final check that the biosimilar product profile and manufacturing process have not changed since the initial litigation under Track Two. Additionally, biosimilar firms would have to place in escrow an amount of money to compensate brand biologics for litigation costs if the biosimilar fails phase 3 trials or if the product or process is changed enough to require additional patents to be litigated.

Of course, there is a risk that a brand would bring a superfluous second litigation based on a small tweak or no change at all in the biosimilar manufacturing process or product profile. Judicial discretion would be necessary to end such cases in summary judgment. Moreover, the biosimilar would more likely launch at risk during such a secondary litigation if the first litigation resolved the uncertainty surrounding patent validity and infringement. This pattern is observed with all biosimilars launching after a district court victory but preceding the CAFC’s ultimate ruling.

Track Two would likely be attractive to many biosimilar firms who wish to enter the market earlier. By entering the market earlier, these biosimilars could benefit from less competition from other biosimilar firms thereby maximizing their revenues. Additionally, patients and payers benefit from earlier market entry because of decreased costs.

This new track would likely need legislation because it would create a new type of patent dance. It would keep much of the original patent dance framework intact, but would allow an alternative pathway that was similar, except for the start of litigation, which would begin at the start of Phase 3 clinical trials instead of after the completion of the clinical trials.

### V.B. Comparison to the European and UK Patent Litigation Systems

In various European countries, including the United Kingdom, generic and biosimilars are permitted to litigate the validity of patents at any time during their product development program. Generic drugs and biosimilars use the same patent litigation pathway. In the UK, Section 72(1) of the Patents Act (the ‘Act’) permits any person to apply for revocation of a patent via the UK Patents Court and it is not necessary to show locus standi. An application for revocation of a patent can be made at any time after notice of grant of the UK patent has been published in the Patents Journal.

In Cairnstores v AB Hassle [2002] F.S.R. 35, the patent owner tried to have the revocation application struck out of court on the grounds that the other party appeared to have lack of trading activities. However, the court held that s.72 of the Act did not require a person applying to revoke a patent to have any interest, commercial or otherwise, in the outcome of the proceeding. Nonetheless, if the patent is eventually found to be valid, the challenger must reimburse the patent owner for the costs spent on the litigation. The policy behind the UK’s position appears to be that it is worthwhile to allocate court resources to remove invalid patents from the public domain. This stands in contrast to the ripeness requirement employed by US Courts, which allows litigation only at a much later stage thereby delaying biosimilar market entry.

UK courts incentivize generics and biosimilar firms to challenge patents at an early stage through the doctrine called ‘clearing the way’, which is an element necessary for injunctive relief. If the generic or biosimilar firm did not attempt to challenge the patent prior to launch, the brand biologic will be more likely to receive a preliminary injunction, thereby preventing biosimilar launch prior to the end of litigation. However, any pre-emptive patent challenge by the generic or biosimilar firm is rewarded by a reduced likelihood of preliminary injunction.

## VI. CONCLUSION

Congress created the BPCIA as a mechanism to get biosimilar products onto the market faster. Unfortunately, the BPCIA has not demonstrated early market entry when compared to generic drugs. The BPCIA worked under the assumption that biosimilar firms would litigate on the same timeline as generic firms. However, this assumption was incorrect. In contrast to generics, biosimilars are required to undergo extensive clinical trials, leading to a substantial delay in market entry. Compounding the problem is delayed litigation because of lack of standing. One straightforward solution is to accelerate the timeline for litigation, allowing biosimilars earlier standing to trigger litigation. Safeguards such as litigation escrow accounts could be used to balance the rights of the brand biologic while incentivizing earlier biosimilar market entry and clearing erroneously granted patents to increase competition. To fulfill the intended objectives of the BPCIA, Congress should create a two-track system that grants biosimilars the option for earlier litigation.

## ROLE OF THE FUNDER

The funder had no role in the design or conduct of the study; collection, management, analysis, and interpretation of the data; preparation, review, or approval of the manuscript; and decision to submit the manuscript for publication.

## ACCESS TO DATA

Drs Tu, Van de Wiele, and Goode had full access to all the data in the study and take responsibility for the integrity of the data and the accuracy of the data analysis.

## FUNDING

Dr Tu research is funded by Arnold Ventures.

## DISCLOSURES

Dr Goode and Mr Turner are employees of Fresenius Kabi. Dr Tu reported consulting for purchasers of lenalidomide containing products outside the submitted work.

## Footnotes

1 Gherghescu I, Delgado-Charro MB. The Biosimilar Landscape: An Overview of Regulatory Approvals by the EMA and FDA. 2021 Pharmaceutics. 13, 48.

2 IQVIA. Biosimilars in the United States 2023–27 (January 2023). https://www.iqvia.com/insights/the-iqvia-institute/reports/biosimilars-in-the-united-states-2023-2027 (accessed June 5, 2023).

3 IQVIA. Biosimilars in the United States 2023–27 (January 2023). https://www.iqvia.com/insights/the-iqvia-institute/reports/biosimilars-in-the-united-states-2023-2027 (accessed June 5, 2023).

4 Tu SS, Lemley MA. *What litigators can teach the patent office about pharmaceutical patents*. Washington University Law Review 99:1673–1731 (2022).

5 Tu SS, Lemley MA. *What litigators can teach the patent office about pharmaceutical patents.*  Washington University Law Review 99:1673–1731 (2022).

6
Van de Wiele V, Raymakers AJN, Kesselheim AS, Tu SS. Changes in Biologic Drug Revenues after Administrative Patent Challenges (Nature Biotechnology in press, 2024).

7 Van de Wiele V, Kesselheim AS, Tu SS. *Biologic Patent Challenges under the America Invents Act*. Nature Biotech 42(3):374–9 (2024).

8 Markurvet D. *Biologics vs. small molecules: Drug costs and patient access*. Medicine in Drug Discovery 9: 100075 (2021).

9
Federal Trade Commission, 2009. Emerging Health Care Issues: Follow-On Biologic Drug Competition: A Federal Trade Commission Report, Washington, DC: Federal Trade Commission.

10 Blackstone, E. & Fuhr Joseph, P., The Economics of Biosimilars, 6(8) Am. Health Drug Benefits 469, 469 (Sep.–Oct. 2013), available at: https://www.ncbi.nlm.nih.gov/pmc/articles/PMC4031732/; Chen et al., An Inflection Point for Biosimilars, McKinsey & Co., (June 7, 2021), https://www.mckinsey.com/industries/life-sciences/our-insights/an-inflection-point-for-biosimilars.

11 21 U.S.C. §355(b)(2)

12 Tu, SS, Kesselheim AS, Ross JS, Gupta R. Revenue, Patents and New Generic Competition for Prescription Drugs in the US. (in manuscript).

13 Beall RF, Darrow JJ, Kesselheim AS. *A Method for Approximating Future Entry of Generic Drugs*. Value in Health 21:1382–9 (2018).

14 Goode R, Feldman WB, Tu SS. *Ancillary Product Patents to Extend Biologic Patent Life.*  Jama 330(21):2117–8 (2023).

15 In the first case to arise out of the BPCIA, Sandoz and Amgen litigated over the early development of a biosimilar version of Amgen’s biologic, Enbrel. In that case, biosimilar maker Sandoz filed an action for declaratory judgment against Amgen. However, Sandoz’s declaratory judgment action posed a jurisdictional issue because the proposed biosimilar was only just entering Phase III clinical trials, and no biosimilar application had been filed. In response, Amgen moved for dismissal. On November 12, 2013, District Judge Maxine Chesney of the Northern District of California dismissed the case for two separate and independent reasons: (1) failure to establish an Article. III ‘case or controversy,’ and (2) failure to abide by the Patent Dance. Sandoz Inc. v. Amgen Inc., 773 F.3d 1274 (Fed. Cir. 2014). See also Section (II)(A).

16 Feldman R. Dance of the Biologics. Berkeley Technology Law Journal (forthcoming). https://papers.ssrn.com/sol3/papers.cfm?abstract_id=4625143 (accessed Apr. 22, 2024).

17 42 USC §262(l)(3)(A).

18 42 USC §262(l)(6)(B).

19 42 USC §262(l)(8)(A).

20 Texas v. United States, 523 U.S. 293, 300 (1998).

21 Sandoz Inc. v. Amgen Inc., 773 F.3d 1274, 1278 (2014).

22 Sandoz Inc. v. Amgen Inc., 773 F.3d 1274, 1277 (2014).

23 Sandoz Inc. v. Amgen Inc., 773 F.3d 1274, 1278 (2014).

24 Sandoz Inc. v. Amgen Inc., 773 F.3d 1274, 1280 (2014). (emphasis added)

25 See Section IV.A. below.

26 Druedah LC, Sporrong SK, Minssen T, Hoogland H, De Bruin ML, Van de Weert M, Almasdotlr, AB. *Inerchangeability of Biosimilars: A Study of Expert Views and Visions Regarding the Science and Substitution*. 17(1) Plos One e026535 (2022).

27 Li F, Vijayasankaran N, Shen A, Kiss R, Amanullah A. *Cell culture process for monoclonal antibody production*. mAbs 2(5): 466–479 (2010).

28 Druedhal LC, Sporrong SK, Minssen T, Hoogland H, DeBruin ML, van de Weert M, Almardottir B. *Interchangeability of Biosimilars: A study of expert views and visions regarding the science and substitution.*  Plos One 17(1):e0262537 (2022).

29 Broer LN, Knapen DG, de Groot PGM, Mol GW, Kosterink EGE, de Vries MNL. *Monoclonal Antibody Biosimilars for Cancer Treatment*. iScience 2024 (in press).

30 Kirsch-Stefan N, Guillen E, Ekman N, Barry S, Knippel V, Killalea S, Weise M, Wolff-Holz E. *Do the outcomes of clinical efficacy trials matter in regulatory decision-making for biosimilars*? Biodrugs 37:855–871 (2023).

31
https://lexmachina.com/ (accessed Oct. 6, 2023).

32
www.clinicaltrials.gov (accessed Oct. 6, 2023).

33
https://www.accessdata.fda.gov/scripts/cder/daf/ (accessed Oct. 6, 2023).

34
https://www.bigmoleculewatch.com/ (accessed Oct. 6, 2023). (also from manufacturer website press releases).

35
https://patents.google.com/ (accessed Apr. 8, 2024).

36
https://www.citeline.com/en/solutions/clinical?utm_source=google&utm_medium=cpc&utm_term=pharmaprojects|&utm_campaign=SO-Clinical-EMEAI&gad_source=1&gclid=CjwKCAiAqNSsBhAvEiwAn_tmxaGHN2LKVQ5kdQljsZhMz6zsosqeB2bsNjBXe42WHKdtaOIEKl4eCRoCZOQQAvD_BwE.

37
https://clinicaltrials.gov/.

38
https://www.accessdata.fda.gov/scripts/cder/daf/index.cfm; https://www.bigmoleculewatch.com/wp-content/uploads/sites/2/2023/12/Big-Molecule-Watch-Biosimilar-FDA-Approvals-Tracker-121523.pdf.

39 Beall RF, Darrow JJ, Kesselheim AS. *A Method for Approximating Future Entry of Generic Drugs*. Value in Health 21:1382–9 (2018).

